# Prediction of blood–brain barrier and Caco-2 permeability through the Enalos Cloud Platform: combining contrastive learning and atom-attention message passing neural networks

**DOI:** 10.1186/s13321-025-01007-2

**Published:** 2025-05-05

**Authors:** Nikoletta-Maria Koutroumpa, Andreas Tsoumanis, Haralambos Sarimveis, Iseult Lynch, Georgia Melagraki, Antreas Afantitis

**Affiliations:** 1 NovaMechanics Ltd, 1070 Nicosia, Cyprus; 2 School of Chemical Engineering, National Technical University of Athens, 157 80 Athens, Greece; 3 Entelos Institute, 6059 Larnaca, Cyprus; 4 School of Geography, Earth and Environmental Sciences, University of Birmingham, Birmingham, UK; 5 Division of Physical Sciences & Applications, Hellenic Military Academy, 166 73 Vari, Greece; 6 NovaMechanics MIKE, 185 45 Piraeus, Greece

**Keywords:** Message-passing neural networks, Attention mechanism, Molecular property prediction, Blood–brain barrier (BBB) permeability, Intestinal barrier permeability, Caco-2 intestinal cells, Molecular contrastive learning, Molecular representation, Web-application, Enalos Cloud Platform

## Abstract

In this study, we introduce a novel approach for predicting two key drug properties, blood–brain barrier (BBB) permeability and human intestinal absorption via Caco-2 permeability. Our methodology centers around a specialized neural network, the atom transformer-based Message Passing Neural Network (MPNN), which we have combined with contrastive learning techniques to enhance the process of representing and embedding molecular structures for more accurate property prediction. These innovative models focus on predicting BBB and Caco-2 permeability -two critical factors in drug absorption and distribution- which fall under the broader scope of ADMET (absorption, distribution, metabolism, excretion, and toxicity) properties. The models are readily accessible online through the Enalos Cloud Platform which offers a user-friendly, AI-powered, ready-to-use web service that significantly streamlines the drug design process, enabling users to easily predict and understand the behavior of potential drug compounds within the human body.

**Scientific Contribution** Our study combines an atom-attention Message Passing Neural Network (AA-MPNN) with contrastive learning (CL), which significantly improves predictive accuracy. Our model leverages self-supervised learning to expand the chemical space used in training and self-attention mechanisms to focus on critical molecular features, enhancing both model accuracy and interpretability. Additionally, the ready-to-use web service based on our model democratizes access to predictive tools for the scientific and regulatory communities.

**Supplementary Information:**

The online version contains supplementary material available at 10.1186/s13321-025-01007-2.

## Introduction

Molecular property prediction, a critical process in drug discovery, involves using models trained on molecules with known and established properties to estimate those of new compounds [1, 2]. This process is vital in the early stages of drug development, as it encompasses the identification of a range of molecular properties, such as lipophilicity, biological activity, and toxicity. Notably, absorption, distribution, metabolism, excretion, and toxicity (ADMET) properties are of paramount importance in drug discovery. These ADMET properties serve as key indicators of a drug candidate's efficacy and safety. Up to 50% of clinical trial failures can be linked to issues with ADMET properties [3], underscoring the importance of these properties to the pharmaceutical industry. Given this context, the efficient and precise evaluation of ADMET properties is critical for streamlining the drug development pipeline.

The quantitative structure–activity/property relationship (QSAR/QSPR) approach is a pivotal technique in the field of computer-assisted drug discovery (CADD) [4]. One of the strengths of the QSAR/QSPR approach is its use of statistical techniques to investigate the relationships between a molecule's chemical structure and its associated properties [5]. This investigation is crucial in developing models capable of accurately predicting how a molecule will behave or react under various conditions. More recent advances within this field have increasingly incorporated machine learning (ML) algorithms into QSAR/QSPR modeling [6], leading to significant improvements, particularly in models focusing on ADMET-related properties. In particular, support vector machines (SVM) and random forest (RF) have become increasingly popular in modeling ADMET properties [7]. These algorithms offer enhanced computational power and sophistication, allowing for more nuanced and precise predictions compared to traditional statistical methods. A critical aspect to ensuring the reliability of the predictions made by QSAR/QSPR models is the accurate molecular representation [8, 9]. The molecule’s representation must encompass all relevant structural information, ensuring that the model can make accurate and useful predictions about the molecule's properties and activities including receptor engagement and protein binding.

The traditional use of fingerprints, such as the Extended-Connectivity Fingerprints (ECFP) [10], and descriptors, while effective, often limits representation to a single dimension, potentially overlooking crucial topological structures of molecules. To address this, numerous studies have shifted focus to 2D graphs for molecular representation [11]. This shift is significant as it enables a more comprehensive capture of molecular structures, offering a broader view of their complex arrangements from 1D to 3D. With the advent and adaptation of deep learning (DL) in processing chemical datasets, there has been a move towards novel forms of molecular representations [12] in which molecules are represented as vectors in high-dimensional, artificially created spaces, called molecular embeddings. These DL models utilize molecular embeddings generated from standard chemical input data, such as string-based representations, with most common the Simplified Molecular Input Line Entry System (SMILES) [13] or chemical graphs [14, 15]. Molecular graphs, preserving rich structural information are often more suitable for molecular property prediction, as well as for tasks in chemical modeling and design [16–19]. There has been substantial application of Graph Neural Networks (GNNs) in molecular property prediction tasks, especially graph convolutional networks (GCNs). Among the GNN variants, the message-passing neural network (MPNN) and the directed MPNN (D-MPNN) stand out as classic methods for aggregating information from molecular graphs [20, 21]. The D-MPNN, proposed by Yang et al. [21], employs a mixed representation involving convolution encoding of molecules and descriptors. This method prioritizes the encoding process and also enhances the model's generalizability, leading to more accurate predictions of molecular properties.

Recent advances have seen the integration of the self-attention mechanism into MPNNs for an enhanced representation of molecular graphs [22–24]. This integration marks a significant shift from traditional models, whereby each atom and bond in a molecular graph is typically given equal significance in determining the predicted outcome. The incorporation of the self-attention mechanism allows the model to specifically focus on those substructures within the molecule that are most critical to the chemical property being predicted. This focus improves the overall accuracy of the model as well as enhancing its interpretability by enabling a clearer understanding of how different atomic or bond structures within a molecule contribute to its overall properties and to a property of interest. This enhanced interpretability is particularly beneficial in drug design, where understanding the relationship between molecular structure and function is crucial. Furthermore, the self-attention mechanism facilitates the visualization of molecular models. Liu et al. [24] proposed integrating both additive attention and scaled dot-product attention at the atomic level into the MPNN framework. Additive attention in this context is used to calculate alignment scores for the hidden states of the encoder and the decoder through feed-forward layers [25]. These alignment scores effectively determine the focus areas of the model, directing attention to specific atoms or bonds that are most informative for the prediction task. Scaled dot-product attention, on the other hand, models interactions between queries and keys using dot products [26]. This mechanism involves a scaling factor that adjusts these results, enabling the model to fine-tune its focus on different parts of the molecular structure. The scaled dot-product attention is particularly adept at capturing complex relationships within the molecular graph, enhancing the model’s ability to learn nuanced representations of molecules.

To accurately predict molecular properties, three critical challenges need to be addressed: 1. Molecules need to be described in a computer-interpretable format to allow computers to process and understand the complex structures of molecules. 2. Molecules need to be transformed into feature vectors, numerical representations that encapsulate the essential characteristics of a molecule. This transformation is a key step in preparing the data for ML models, as it translates complex molecular information into a format that algorithms can process and learn from; and 3. A predictive model needs to be trained with a large dataset of labeled molecules. The first two challenges, molecular representation and featurization are discussed above, with several approaches describing best each problem. However, the number of available labeled molecules for training is often insufficient for the needs of molecular prediction benchmarks. When ML models are trained on limited labeled data, there is a risk of overfitting, where the model performs well on the training data but poorly on new, unseen data, particularly if the new molecules are structurally different from those in the training set. To mitigate the risk of overfitting, self-supervised learning (SSL) has emerged as a promising solution [27–29]. SSL techniques are now being applied to pretrain GNNs by utilizing the vast amounts of available unlabeled molecular data and can significantly improve the performance of models in predicting molecular properties [27, 28, 30, 31].

Contrastive learning (CL), a prominent SSL algorithm, is extensively utilized for learning representations by differentiating between similar and dissimilar samples. The essence of CL is its ability to discriminate between pairs of samples that are jointly sampled (viewed as similar) and those that are independently sampled (viewed as dissimilar) [32]. A critical application of CL is in Graph Contrastive Learning (GCL), where the goal is to learn unsupervised representations for molecular graphs [27]. This approach is particularly valuable in the domain of computational chemistry, where understanding the intricate structures and properties of molecules is essential. In GCL, positive samples, representing similar molecular structures, can be constructed using various graph augmentation techniques, such as node or edge dropping, shuffling, or attribute masking [27]. Each augmentation creates a slightly different version of the original molecular graph, providing a basis for the model to learn the essential features of the molecules. In this way, the model learns to identify and emphasize the key features of the molecules that remain consistent across various augmentations, thereby gaining a deeper understanding of the inherent properties of the molecules.

**Fig. 1:**
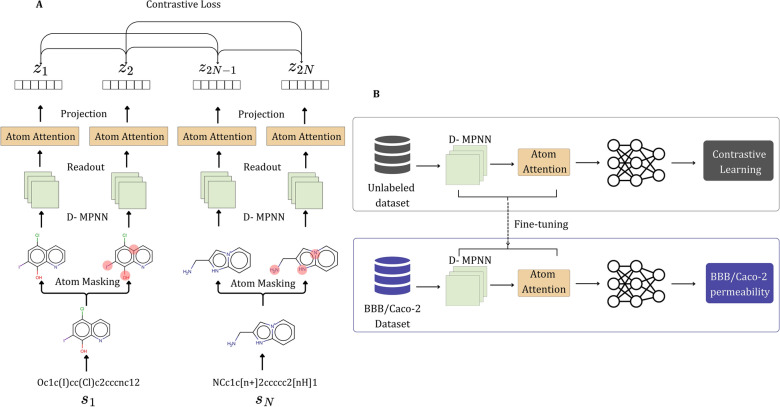
Illustration of the framework of the proposed method for molecular property prediction. **A** Pretraining module that consists of four components: atom masking for graph augmentation, D-MPN encoder, multi-head atom attention layer, and contrastive loss. **B** The entire framework: the atom attention D-MPN encoder is pretrained using a large unlabeled dataset and the representations are projected through a multilayer perceptron projection head. Contrastive loss is utilized to maximize the agreement between positive pairs. As a result, the atom attention encoder learns representative features of the molecules. The pretrained parameters are transferred to a new model and are fine-tuned for a specific molecular property prediction task. An FFN is randomly initialized and trained to predict the specific molecular property

In this work, we incorporate atom-attention MPNN (AA-MPNN) with molecular CL to boost the performance of predictive models [24, 27], focusing on prediction of molecules that can penetrate the blood–brain barrier (BBB) or be adsorbed through the intestinal barrier. This integration focuses on using additive attention and scaled dot-product attention to highlight critical substructures within the molecular graph, resulting in generation of more informative and detailed molecular representations. The additive and scaled dot-product attention mechanisms selectively concentrate on key areas of the molecular graph, thereby improving the model's ability to identify and process significant structural details linked to barrier penetration or adsorption. The proliferation of available molecular data has facilitated the development of a CL framework designed specifically to improve the learning of molecular representations and thus the prediction of molecular properties. At the core of this framework is the atom-attention MPN encoder, which is initially pretrained on a substantial dataset of unlabeled molecules. This pretraining phase is critical for the model to learn general representations of molecular structures without the need for labeled data. A key strategy employed in this work involves the creation of positive molecule graph augmented pairs, a technique proposed by Wang et al. [27]. This technique, known as atom masking, involves selectively hiding certain atoms within the same molecule to generate variations of the molecular graph. These variations serve as positive pairs for CL, enabling the model to learn by comparing these positively paired graphs against negatively paired ones from different molecular structures. Following the pretraining, the model employs a contrastive loss function to learn representations based on the contrasts between these positive and negative molecular graph pairs. After this phase, the model is further refined with a feed-forward network (FFN). This FFN is trained using specific datasets for downstream molecular property prediction tasks. By employing this method, we demonstrate that pretraining on large, diverse chemical datasets significantly improves the performance of models in predicting molecular properties that are associated with biological barrier interaction. This work illustrates a sophisticated approach to enhancing molecular property prediction models (in this case biological barrier penetration or adsorption across a barrier) through a combination of atom-attention MPNNs, molecular CL, and strategic data augmentation techniques. The effectiveness of this approach is underlined by its ability to accurately predict BBB permeability and human intestinal absorption, two critical aspects of drug absorption and distribution. The entire workflow of this research is detailed in Fig. 1, showcasing the comprehensive process from data preparation to model training and application.

## Materials and methods

### Datasets

#### Blood–brain barrier permeability

Clinical experiments to determine the BBB permeability of compounds are both time-consuming and labor-intensive. The BBB serves as a primary defense mechanism, shielding the brain from exposure to potentially toxic substances. Due to its restrictive nature, most compounds do not successfully penetrate the BBB membrane. Evaluating BBB permeability is crucial for assessing the potential toxicity of new pharmaceuticals. Over the years, several QSPR models have been developed to predict BBB permeation. For this study, a comprehensive dataset of 7,807 compounds, categorized based on their BBB permeability (BBB + or BBB-), was compiled from the literature [33] to train the predictive model. The datasets used in this study were prepared using ChEMBL standardization and neutralization procedures, ensuring consistency in molecular structures and their representations [33]. Note that the focus of the dataset is on the chemical properties and provides no information on the nature of the exposures (in vitro, in vivo) nor any details of the comparability of the barrier models utilized. Our future work includes an exploration of the impact of the BBB models themselves.

#### Caco-2 cell line permeability

The Caco-2 cell line model is a standard method for assessing the in vitro membrane permeability of drugs. This method, however, requires a costly and time-consuming culturing process, prompting the need for a more rapid and accurate alternative to evaluate oral drug permeability. A significant literature dataset of data on the Caco-2 cell line permeability [34] was utilized. Any compounds with unclear SMILES codes or permeability values outside the range of ${10}^{-3.5} cm\cdot {s}^{-1}$ to ${10}^{-8} cm \cdot {s}^{-1}$, which are considered potential unreliable [35], were excluded. Furthermore, salts and solvents were removed, and the compounds were standardized. To determine an objective threshold for classification, the remaining compounds were then processed using the k-Means clustering algorithm to categorize them into two groups: permeable and non-permeable, based on their permeability values [36]. Compounds with permeability values less than or equal to -5.5 logPapp units $({10}^{-5.5} cm\cdot {s}^{-1})$ were classified as non-permeable, while those with values greater than -5.5 logPapp units ${(10}^{-5.5} cm\cdot {s}^{-1})$ were considered permeable. The resulting threshold aligns well with empirical cutoffs found in the literature, where previous studies have used the threshold of logPapp = -5.1 to distinguish high and poor permeability [37]. Furthermore, Metoprolol with a logPapp value of -4.7 is considered by the FDA as the high permeability class boundary. Similarly, in another study, permeability values were classified as follows, logPapp < -6 for low permeability, -6 < logPapp < -5 for low-moderate permeability, -5 < logPapp < -4.7 for moderate-high permeability and logPapp >  = -4.7 for high permeability [38]. As a result, the threshold logPapp = -5.5 provides a reasonable approximation for distinguishing compounds between low and high permeability. More information regarding the distribution of permeability values is available in Supplementary Information. The final modeling dataset comprised 1,827 compounds, of which 1,127 (62%) were classified as permeable.

#### Pretraining dataset

For pretraining the atom-attention MPN encoder, we used 250,000 unique unlabeled molecule SMILES collected from the ZINC15 database [39]. The ZINC15 database was chosen for its extensive collection of commercially available biomolecular compounds, which includes natural products, metabolites, and FDA-approved drugs, making it highly relevant for our predictive modeling needs. The ZINC15 subset employed for pretraining was retrieved from Fang et al. [40, 41], comprising drug-like and easily synthesizable molecules with diverse chemical scaffolds, as shown in Supplementary Information Figure S2, S3, Table S2. As a result, it captures a broad segment of the chemical space, regarding drug-like molecules. These features make it a robust dataset for pretraining our model. To ensure there was no data leakage, we verified that molecules in the BBB and Caco-2 cell line datasets were not present in the ZINC15 pretraining set. We performed a structural similarity analysis between the BBB and Caco-2 cell line dataset and the ZINC15 database using Tanimoto similarity. By setting a similarity threshold of 85%, we found no compounds in the Caco-2 dataset that were similar to those in the ZINC15 subset. The BBB dataset contained only four compounds with a similarity greater than 85% to ZINC15, none of which were identical to any compounds in the ZINC15 subset. A visualization of the maximum similarities between the datasets is available in Supplementary Information Figure S4. This analysis confirms that there is a minimal overlap between the datasets. To facilitate effective model training and evaluation, we divided the pretraining dataset into a training and a validation set using a 90:10 split. This distribution allowed training of the model on a substantial portion of the data while reserving a smaller segment for validation purposes, ensuring that the model is tested on unseen data, thereby evaluating its predictive performance accurately.

### Directed message-passing neural encoder

Each SMILES representation was converted into a directed graph. Conceptually, a molecule can be considered as a graph consisting of a set of atoms (nodes) and a set of bonds (edges) which represent interactions between each pair of adjacent atoms. A graph $G=\left(V,E\right)$ defines the connectivity relations between a set of nodes $\left(V\right)$ and a set of edges $\left(E\right)$. Thus, the graph-based representation encodes properties or relationships of atoms and bonds locally with a collection of atom and bond feature vectors. The D-MPNN [21] framework involves two key phases to extract global features: the message-passing phase and the readout phase.

During the message-passing phase, the MPNN gradually integrates information from distant atoms by extending through bonds radially. In each message-passing step $t$ $\left(1\le t\le T\right)$, over T iterations, the message ${m}_{vw}^{t}$ and the hidden state ${h}_{vw}^{t}$ from atom $v$ given node features ${x}_{v}$ and edge features ${e}_{vw}$ is updated as follows:

1 $${m}_{vw}^{t}=\sum_{w\in N\left(v\right)}{M}_{t}({h}_{v}^{t-1}, {h}_{wv}^{t-1}, {e}_{vw})$$

2 $${h}_{vw}^{t}={U}_{t}\left({h}_{vw}^{t-1}, {m}_{vw}^{t}\right)=\tau ({h}_{v}^{0}+{W}_{h}{m}_{vw}^{t})$$

where ${M}_{t}$ is a message function, ${U}_{t}$ is an atom update function and ${W}_{h}$ is the learn weight matrix. Node features ${x}_{v}$ are derived from atom type, the number of bonds the atom is involved in, formal charge, chirality, number of bonded hydrogens and atom’s hybridization. Edge features ${e}_{vw}$ are derived from bond type, ring status and stereochemistry. The detailed descriptions of node and edge features are displayed in Supplementary Information Table S3 and S4, respectively. These features provide the essential information needed to model the molecular interactions effectively, thereby allowing the neural network to generate accurate predictions based on the molecular structure.

In the readout phase, all hidden representations of nodes are aggregated to a global representation for the entire graph as follows:

3 $${H}_{v}=\sum_{v\in G}{h}_{v}$$

4 $$y=R(\left\{{H}_{v}|v\in G\right\})$$

where ${h}_{v}$ is summed for a total $T$ steps and the readout function $R$ is used to aggregate the characteristics $y$ of the molecule.

### Atom transformer-based MPNN

The transformer is a relatively new DL approach that uses the attention mechanism to differentiate the importance of each part of the input data [26]. A self-attention layer takes the input hidden matrix $H\in {\mathbb{R}}^{N\times d}$, where $d$ is the hidden dimension and $N$ is the number of entries. The input is associated with three matrices, a query matrix $(Q=H{W}_{Q})$, a key matrix $(K=H{W}_{K})$, and a value matrix $(V=H{W}_{V})$, where ${W}_{Q}, {W}_{K}, {W}_{V}$ are the parameter matrices. The self-attention in the Transformer is computed as follows for a single-head self-attention and a multi-head self-attention by multiplying with a parameter matrix ${W}_{o}$:

5 $$single \,head= Attention\left(Q,K,V\right)=softmax(\frac{Q{K}^{T}}{\sqrt{d}})V$$

6 $$multi\,head\left(Q,K,V\right)=Concat\left(hea{d}_{1},\dots ,hea{d}_{h}\right){W}_{o}$$

Molecules represented by SMILES are converted into molecular graphs which contain atom features ($${x}_{v})$$ and bond features ($${e}_{vw})$$ as one hot encodings. Following the D-MPNN architecture, which consists of a message-passing phase through directed bonds and a readout phase, each bond is initialized with two feature vectors, for bidirectional bond messages. The atom features and bond features are first concatenated and passed through a weight matrix $${W}_{i}$$ and an activation function, producing the initial bond hidden state $${h}_{vw}^{0}$$ (Table 1: Initialization). In the message-passing phase, the bond message at each iteration $$t$$ is updated by summing all the previous hidden states $${h}_{kv}^{t-1}, k \epsilon Neighbor(v)$$ except the hidden state of the opposite direction. The bond message $${m}_{vw}^{t}$$ passes through a weight matrix $${W}_{h}$$ and is then concatenated with the initial bond hidden state $${h}_{vw}^{0}$$ and is fed into an activation function to generate the hidden state $${h}_{vw}^{t}$$ (Table 1: Bond Embedding Phase). After $$T$$ message-passing iterations, the bond hidden states are aggregated and concatenated with atom features, and are transformed by a weight matrix ($${W}_{0}$$) and an activation function producing the message of each atom $${m}_{v}$$.

**Table 1:** Pseudocode of the transformer-based MPNN presented herein

*Initialization*	
For each atom $v$ in molecule $G$: For each atom $w$ in molecule $Neighbor(v):$ ${h}_{vw}^{0}\leftarrow ReLU({W}_{i}Concat({x}_{v},{e}_{vw})$	
*Bond aggregation*	
While $1\le t\le T:$ For each atom in molecule $G$: For each atom $w$ in molecule $Neighbor(v):$ ${m}_{vw}^{t}\leftarrow {\sum }_{k\epsilon Neighbor(v)}{h}_{kv}^{t-1}-{h}_{wv}^{t-1}$ ${h}_{vw}^{t}\leftarrow ReLU({h}_{vw}^{0}+{W}_{h}{m}_{vw}^{t})$	
*Atom aggregation*	
For each atom $v$ in molecule $G:$ ${m}_{v}\leftarrow ReLU\left({W}_{0}Concat\left({x}_{v},{\sum }_{w\epsilon Neighbor\left(v\right)}{h}_{vw}^{T}\right)\right)$ ${h}_{v}\leftarrow AtomAttention\left({m}_{v}\right)+{m}_{v}$	
*Molecule aggregation*	
$h\leftarrow {\sum }_{v\in G}{h}_{v}$ $y\leftarrow FFN(Concat(h, {h}_{f}))$	

**Fig. 2:**
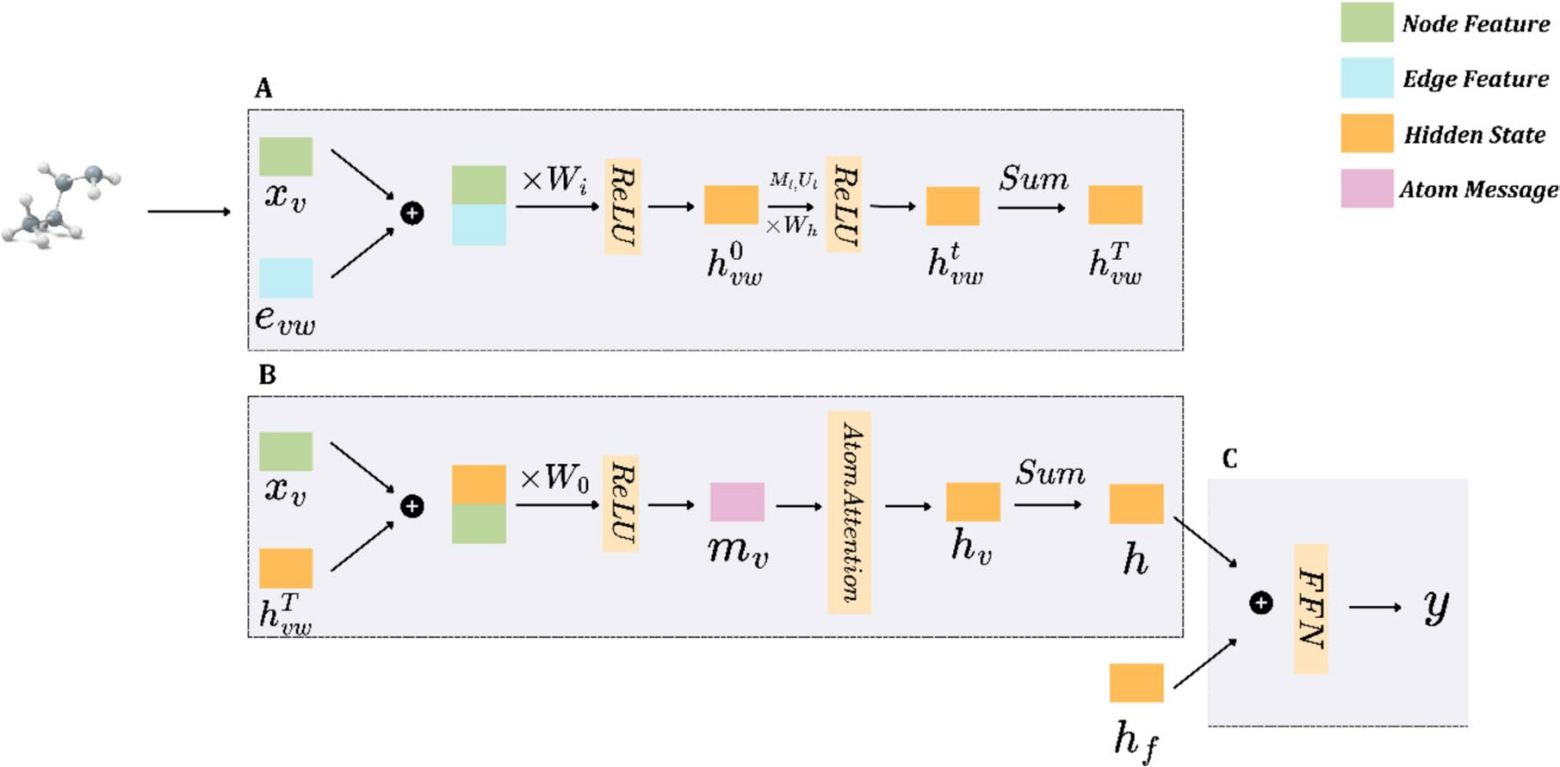
Diagram of the transformer-based MPNN. The framework consists of **A** a D-MPNN, **B** an atom attention multi-head transformer, and **C** a FFN to predict the property of interest (in this case the molecule’s ability to cross a biological barrier). Each component in this figure represents different vector representations within the model, as defined in the pseudocode in Table 1

A multi-head attention layer is then added during the readout phase to identify the relationship between the substructure and its contribution to the target property. The atom attention layer takes as input a hidden matrix ${H}_{a} \epsilon {\mathbb{R}}^{M\times d}$, which is the aggregation of atom messages, where $M$ is the number of atoms and $d$ is the hidden dimension (Table 1: Atom Embedding Phase). After aggregating the atom messages over the molecule, the molecular vector is concatenated with the extended-connectivity fingerprint (ECFP) and then entered into an FFN. The final output of the model is returned by a two-layer FFN, predicting the property of interest (Table 1: Molecule aggregation). The architecture of the proposed Transformer-based MPNN is shown in Fig. 2.

### Contrastive learning

CL leverages SSL on extensive amounts of unlabeled data, enabling models to capture rich semantic information about molecules [42]. This method involves learning representations by contrasting positive example data pairs with negative example data. In the molecular comparative learning scheme implemented here, a batch of $N$ molecules was randomly selected and their positive samples generated, resulting in $2N$ molecular samples. Drawing on the methodologies developed by Chen et al. [43], You et al. [44], and Fang et al. [40], our objective is to minimize the similarity within each sample of positive pairs while maximizing the dissimilarity between the negative pairs. In the representation space, our goal is for positive pairs to be as close as possible and for negative pairs to be as distant as possible. To achieve this, the cosine similarity function was employed to measure the distance or similarity between two vector representations ${z}_{1}$, ${z}_{2}$ in the projection space, defined as:

7 $$si{m}_{({z}_{1},{z}_{2})}= \frac{{z}_{1}^{T}{z}_{2}}{\Vert {z}_{1}\Vert \cdot \Vert {z}_{2}\Vert }$$

The CL framework utilizes a normalized temperature-scale cross-entropy loss (NT-Xent loss) [43]. The training objective for graph ${G}_{i}$ and ${G}_{i}{\prime}$ is defined as:

8 $${\mathcal{L}}_{i,j}=-\text{log}\frac{{e}^{sim({z}_{i},{z}_{i}{ {^{\prime } } })/\tau }}{\sum_{j=1}^{N}\left({e}^{sim({z}_{i},{z}_{j}{ {^{\prime } } })/\tau }+ {e}^{sim({z}_{i}{ {^{\prime } } },{z}_{j})/\tau }\right)}$$

where, $\tau$ denotes the temperature parameter and $sim({z}_{1},{z}_{2})$ is the cosine similarity.

For molecular graph data augmentation, various approaches have been proposed, with GCL outlining a comprehensive graph learning scheme for learning unsupervised representations of molecular graphs [44]. In our study, we utilized atom masking to create a positive pair of samples, masking atoms in the molecule randomly with a ratio of 25%. When an atom is masked, its atom feature ${x}_{v}$ is replaced by a mask token $m$, which is distinct from any other atom features in the molecular graph. This method of atom masking allows the model to learn the intrinsic features of molecules by focusing on the unmasked portions of the molecule, enhancing its ability to generalize from partial data.

### Model training and evaluation

For the model training, each atom in the molecular graph is characterized by specific features such as atomic number, degree of freedom, formal charge, chirality, the number of bonded hydrogens, and the atom's hybridization. Similarly, each bond within the molecule is described by its type, stereochemistry, and presence in a ring. Both the MPNN and the atom transformer were implemented using Python and PyTorch version 2.0 [45]. To enhance model performance, hyperparameters were optimized using Bayesian Optimization, applying consistent parameters across 20 epochs and 20 iterations [46].

**Fig. 3:**
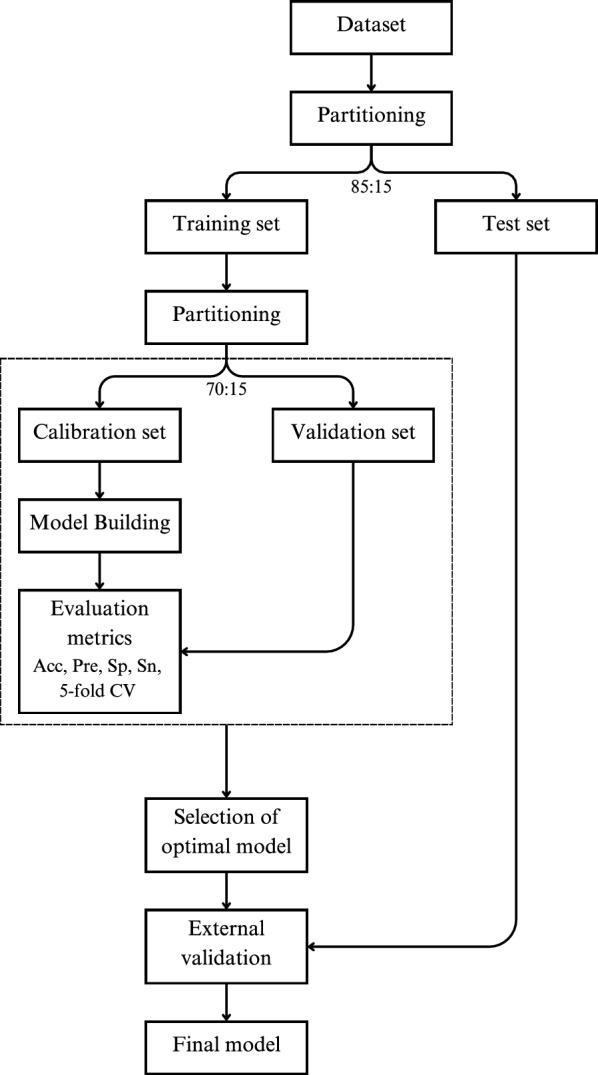
Analysis workflow, model implementation, as presented here for an atom transformer-based MPNN model

The model was optimized for both datasets, BBB and Caco-2 cell line, focusing on four hyper-parameters as detailed in Table 2. Optimization was carried out using the Adam optimizer with learning rates $lr$ ranging from ${10}^{-4}$ to ${10}^{-3}$. The optimal parameters were selected based on the highest performance scores obtained on the validation set during the training phase. The datasets were divided using a random split approach, allocating 85% for training and 15% for testing. The training subset was further divided into calibration and validation sets with a split ratio of 70:15. Five-fold cross-validation (CV) was performed on these partitioned data splits, and reported as the mean and standard deviation of the evaluation metrics. The final evaluation of the selected model was conducted using the test set to verify the model's efficacy on predicting unseen data. This rigorous validation scheme, as depicted in Fig. 3, ensures that our model is robust and reliable, suitable for practical applications in predicting molecular properties, in this case biological barrier crossing.

**Table 2:** Bayesian Optimization for Hyperparameter tuning

Hyperparameter	Values
Message-passing iteration	2, 3, 4, 5, 6
Batch size	128, 256, 512
Dropout probability	[0.0, 0.4] (Interval: 0.05)
Number of layers in FFN	2, 3

During the pretraining phase, positive pairs were created by masking 25% of the total atoms in each molecule, a technique aimed at reducing the normalized temperature-scaled cross-entropy loss (NT-Xent) loss between these pairs. For downstream tasks, the molecular vector $h$ is concatenated with the molecular fingerprint ${h}_{f}$ and an FFN is initialized atop the atom-attention MPN encoder (as shown in Fig. 2). In this setup, which focuses on classification tasks, binary cross-entropy loss is utilized to gauge model performance.

### Evaluation protocols

The performance of our model was assessed primarily using the area under the receiver operating characteristic curve (ROC-AUC) where higher values signify better performance. This metric is critical as it measures the ability of the model to distinguish between classes effectively. In addition to ROC-AUC, we employ several other metrics to provide a comprehensive evaluation, including accuracy, precision, sensitivity and specificity (Supplementary Information Table S5).

## Results and discussion

Empirical evaluation of our proposed atom-attention MPNN (AA-MPNN) model is presented and its effectiveness is demonstrated. Through rigorous testing and analysis, the capabilities and performance improvements brought about by integrating atom-attention mechanisms into the MPNN framework are highlighted. By assessing the model across various metrics and scenarios, its utility in practical applications and its contribution to the field of molecular property prediction can be better understand.

### Main results on molecular property prediction

The first analysis considers whether the proposed CL approach performs better than the non-pretrained QSPR model. To assess the effectiveness of CL, we first evaluated the performance of the models using fivefold cross-validation on the training data. Table 3 summarizes the average performance metrics across all folds. Models incorporating CL consistently outperformed the non-pretrained models, demonstrating improved evaluation metrics. More specifically, for BBB permeability prediction, the model without CL achieved an average ROC-AUC of 0.944 + 0.007 and an average accuracy of 0.874 + 0.008. When using CL, the model achieved an ROC-AUC of 0.951 ± 0.006 and accuracy of 0.882 ± 0.013. For Caco-2 permeability, the model without CL obtained an average ROC-AUC of 0.905 + 0.022 and an accuracy of 0.842 + 0.024, while model with CL achieved ROC-AUC of 0.919 ± 0.019 and accuracy of 0.848 ± 0.032. These results highlight the benefits of CL, consistently leading to enhanced predictive performance across both permeability tasks. More details about each fold are available in Supplementary Information Figure S5 and Figure S6, highlighting the stability of these findings across data splits. During fine-tuning for both downstream tasks, we optimized the hyper-parameters defined in Table 2, to find the best performing setting on the validation set and present the results on the test set.

**Table 3:** Evaluation metrics for BBB and Caco-2 permeability on fivefold cross-validation for models with and without CL

	BBB Permeability		Caco-2 Permeability	
Metrics	Without CL	With CL	Without CL	With CL
ROC-AUC	0.944 + 0.007	0.951 ± 0.006	0.905 + 0.022	0.919 ± 0.019
Accuracy	0.874 + 0.008	0.882 ± 0.013	0.842 + 0.024	0.848 ± 0.032
Precision	0.879 + 0.004	0.892 ± 0.009	0.850 + 0.026	0.855 ± 0.025
Sensitivity	0.929 + 0.013	0.927 ± 0.018	0.813 + 0.031	0.897 ± 0.037
Specificity	0.776 + 0.009	0.803 ± 0.015	0.746 + 0.040	0.756 ± 0.062

**Fig. 4:**
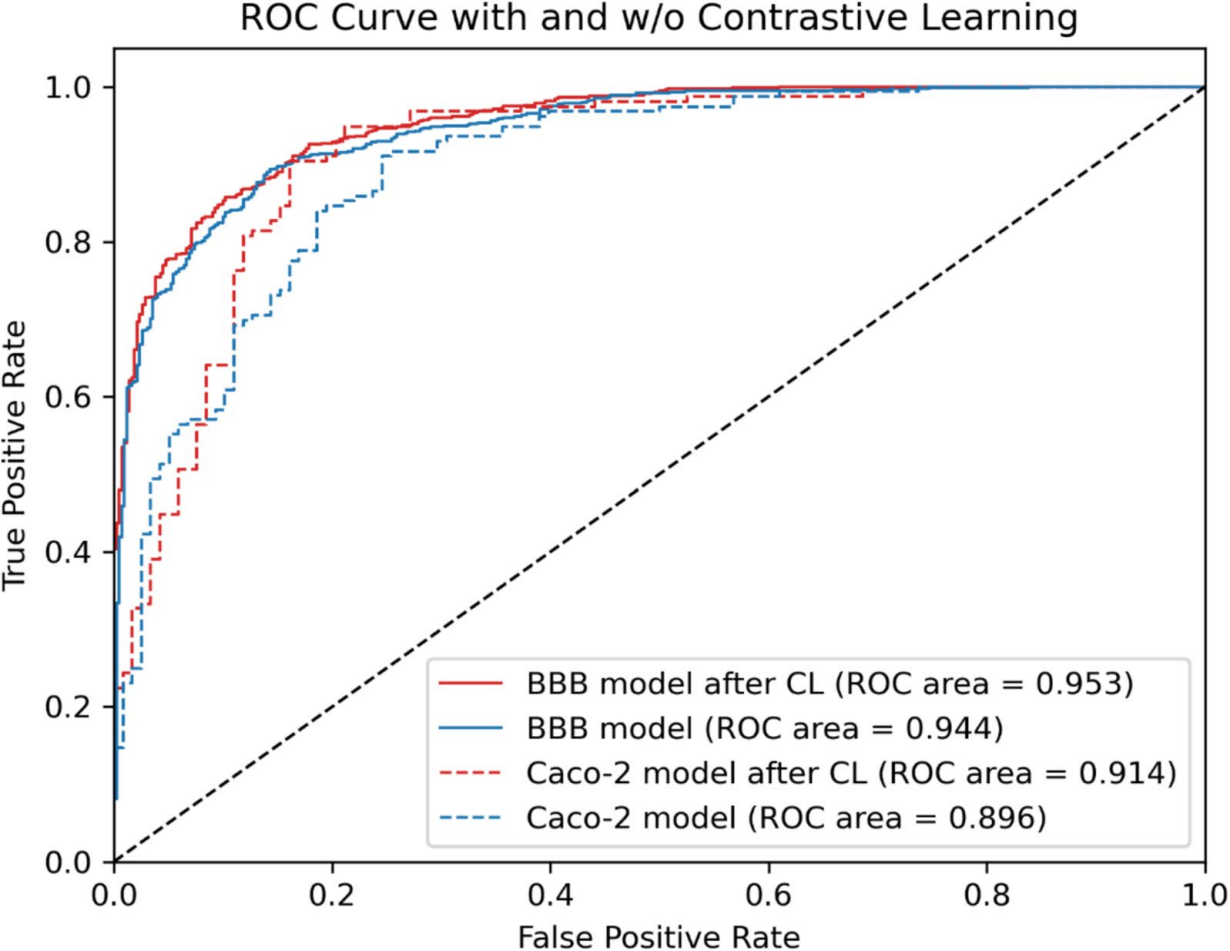
ROC curves of the models (BBB model, Caco-2 cell line model) with CL and without CL, and their respective AUCs

Following model selection based on cross-validation, we evaluated the performance on external test set (Table 4). Consistent with CV results (Table 3), the pretrained models outperformed the non-pretrained models, reinforcing the advantages of pretraining for molecular representation learning. The comparison of the ROC curves (Fig. 4) between model with and without CL also indicates the better performance of the model with CL.

**Table 4:** Evaluation metrics for BBB and Caco-2 permeability prediction for test set with and without CL

	BBB Permeability		Caco-2 Permeability	
Metrics	Without CL	With CL	Without CL	With CL
ROC-AUC	0.944	0.953	0.896	0.914
Accuracy	0.868	0.885	0.828	0.872
Precision	0.866	0.898	0.801	0.846
Sensitivity	0.939	0.926	0.929	0.949
Specificity	0.740	0.812	0.695	0.771

By leveraging the atom-attention MPNN model enhanced with CL, our approach significantly improves the accuracy of molecular property predictions, which is pivotal for computational drug discovery. The integration of sophisticated ML techniques, such as CL, enables a more refined representation of molecular structures, essential for identifying potential drug candidates with desired properties. Furthermore, the enhancements observed in ROC-AUC, accuracy, precision, sensitivity, and specificity substantiate the atom-attention MPNN with CL model's capability to effectively distinguish between permeable and non-permeable molecules—crucial for reliable drug screening processes. These metrics illustrate the model's proficiency in predicting interactions of molecules with biological barriers, such as the blood–brain barrier or intestinal walls, which are critical considerations in the pharmacokinetics of drug design.

**Fig. 5:**
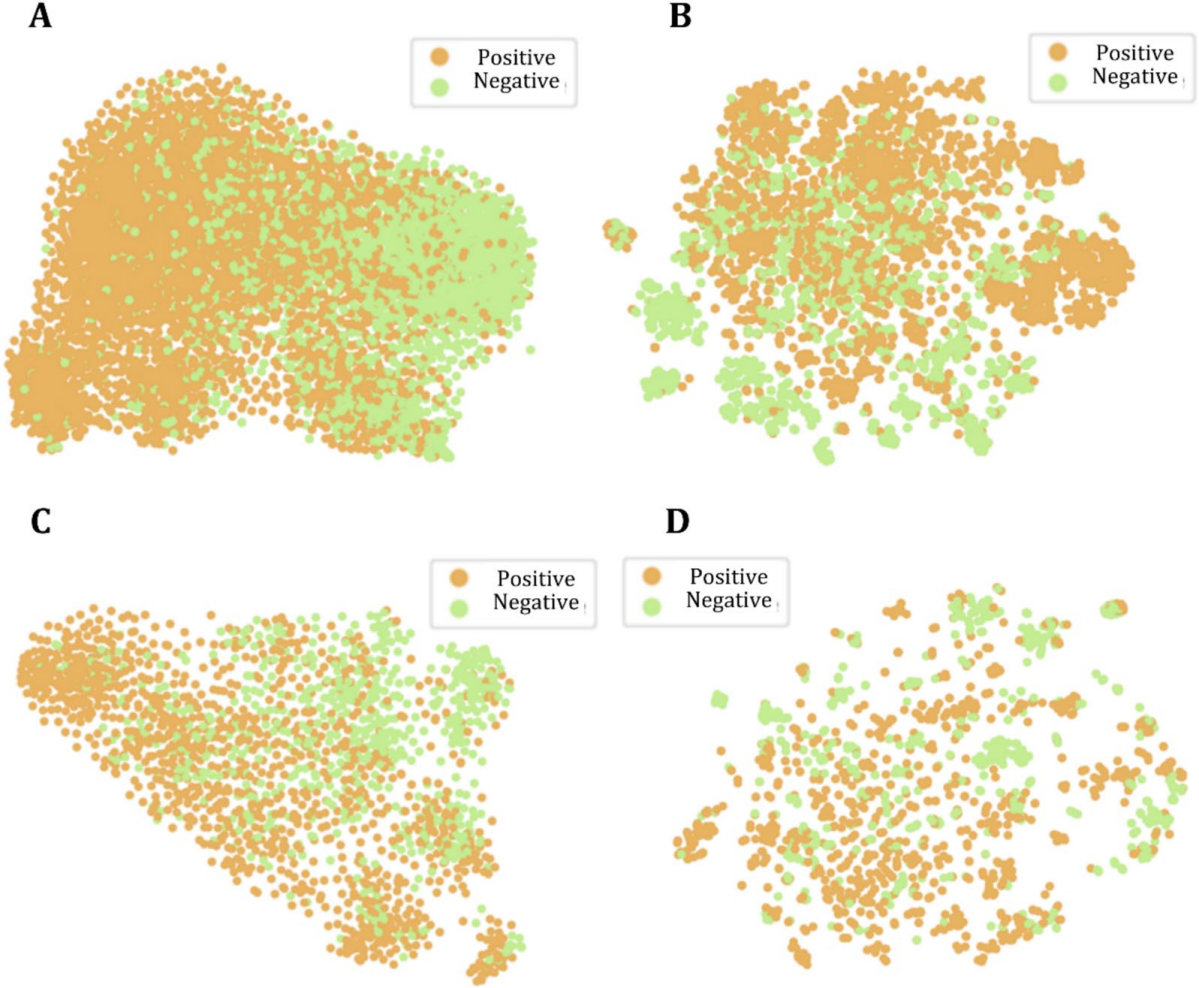
Investigation of molecular representation based on t-SNE analysis. **A** t-SNE analysis on non-pretrained MPN encoder on BBB dataset. **B** t-SNE analysis on pretrained MPN encoder on BBB dataset. **C** t-SNE analysis on non-pretrained MPN encoder on Caco-2 cell line dataset. **D** t-SNE analysis on pretrained MPN encoder on Caco-2 cell line dataset

The t-distributed Stochastic Neighbor Embedding (t-SNE) technique [47] was also applied to visualize the molecular representations learned by the MPN encoder. As a dimensionality reduction tool, t-SNE excels in visualizing high-dimensional data in 2D space. Through this method, molecules with similar properties in high-dimensional space are mapped to nearby points in low-dimensional space, while those with dissimilar properties are positioned further apart. As depicted in Fig. 5, t-SNE analysis on the BBB and Caco-2 cell line datasets effectively compares the results of the applied pretraining strategy against non-pretrained models. Post-pretraining, the model's representations show distinct clustering characteristics, with molecules bearing similar labels clustering more closely. This underscores the efficacy of pretraining in boosting the accuracy of downstream classification tasks. Such visual insights not only confirm the benefits of the pretraining but also shed light on the molecular diversity managed by the model, aiding in the exploratory analysis of molecular structures which may lead to the identification of new biomarkers or therapeutic targets, in this case for neurodegenerative or other brain-related conditions and for oral delivery via crossing of the intestinal barrier. The consistent performance across multiple folds of CV highlights the robustness and generalizability of our model. This reliability is crucial in pharmaceutical research, where predictive models must consistently identify potential efficacy and safety of compounds before proceeding to expensive clinical phases. The robust performance reassures users of the utility of our model in real-world settings which are characterized by a wide variability in molecular data.

### Chemical scaffold and chemical space analysis of datasets

**Fig. 6:**
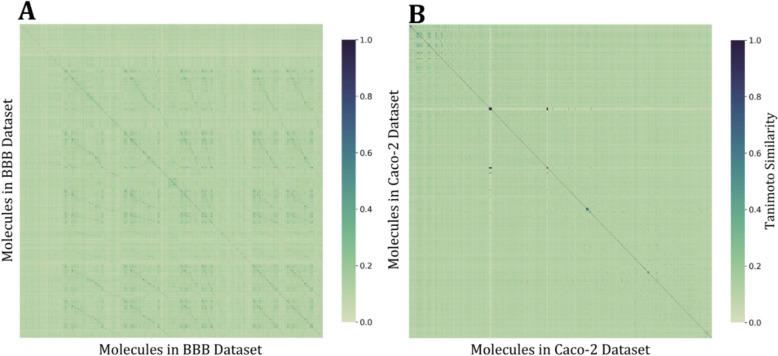
Tanimoto Similarity Matrix of ** A**. BBB dataset and** B**. Caco-2 dataset

The quality of the data is crucial for the development of a robust predictive model. To assess the chemical diversity and distrubution of data in the chemical space, the Murcko scaffold approach was utilized. The Murcko scaffold is the unique ligand and ring system remaining after removing all substituents [48]. Using this method, we analyzed the chemical diversity of the BBB and Caco-2 datasets. The BBB contains 2,129 unique Murcko scaffolds, whereas the Caco-2 dataset comprises of 1,027 unique Murcko scaffolds. Notably, 67% of scaffolds in the BBB dataset and 85% of scaffolds in the Caco-2 dataset are generated by only one or two molecules, highlighting the diversity within the datasets. Furthermore, the average Tanimoto similarity of each compound in each dataset was calculate to visualize the chemical diversity and chemical space distribution. The similarity heatmaps presented in Fig. 6 illustrate these distributions, providing insights into the structural diversity across the datasets.

The presence of a large number of unique scaffolds, along with the distribution of molecular structures, indicates that the datasets cover a broad and diverse chemical space, ensuring a comprehensive representation of different structural categories. To explore the impact of scaffold diversity on model performance, we evaluated our model only on test compounds whose scaffolds were absent from the training dataset. On these compounds, our BBB permeability model achieved an AUC of 0.795, while the Caco-2 permeability model achieved an AUC of 0.897 for compounds with scaffolds absent in the training dataset.

### Comparison of model performance

Our findings align with and extend the results of previous studies in this domain. For instance, Hamzic et al. improved the prediction of brain penetration by integrating in vitro experimental data as auxiliary tasks resulting in Matthew’s correlation coefficient (MCC) of 0.66, sensitivity of 0.96 and specificity of 0.63 [49]. Kumar et al. introduced a novel approach, the classification read-across structure activity relationship (c-RASAR) to improve BBB permeability prediction, resulting in AUC of 0.92 and sensitivity of 0.88 [50]. While we cannot directly compare our results due to different data partitioning and splitting techniques, our model achieved an AUC of 0.95, sensitivity of 0.93 and specificity of 0.81, demonstrating strong performance in predicting BBB permeability. Additionally, recent studies for Caco-2 permeability prediction have explored both QSPR-based and DL approaches. For instance, a supervised recursive ML approach was used for predicting Caco-2 permeability resulting in models with root mean squared error (RMSE) values between 0.43 and 0.51 [38]. Similarly, a multi-embedding-based synthetic network has shown superior predictive performance across various pharmacokinetic properties, including membrane permeability, with a mean absolute error of 0.41 [51]. While these methods provide high predictive performance, our atom-attention MPNN model offers additional advantages by leveraging CL and self-attention mechanisms to enhance interpretability. By identifying critical atomic contributions to permeability, our approach enables a deeper understanding of molecular features influencing barrier crossing, thereby facilitating rational drug design.

Furthermore, the results of our method were compared with commonly used ML models for property prediction. In each QSPR task, we built RF, SVM, FFN and AA-MPNN with CL. We used ECFP as inputs to RF, SVM and FFN to compare with AA-MPNN-CL which also concatenated the molecular vectors with ECFP in the last layers of the model. In addition, while AA-MPNN-CL incorporates ECFP features in its final FFN, we also evaluated a version of AA-MPNN-CL trained without ECFP to assess the impact of molecular representations learned by atom-attention message passing and contrastive learning alone. The results, summarized in Table 5, indicate that AA-MPNN-CL without ECFP still achieves strong performance (ROC-AUC of 0.938 ± 0.008 of BBB permeability and ROC-AUC of 0.904 ± 0.019 for Caco-2 permeability) compared to other methods, demonstrating the predictive power of the learned representations. However, incorporating ECFP further enhances predictive accuracy, with AA-MPNN-CL with ECPF achieving ROC-AUC of 0.951 ± 0.006 for BBB permeability and 0.919 ± 0.019 for Caco-2 permeability. These results suggest that atom-attention message passing and contrastive learning effectively captures molecular information compared to single fingerprint-type representations. By combining these molecular vectors with ECFP fingerprints, enhances performance by leveraging complementary structural features.

**Table 5:** Comparisons of performance with ML models on QSPR tasks

Dataset	Method	ROC-AUC
BBB permeability	RF-ECFP	0.911 ± 0.004
	SVM-ECFP	0.901 ± 0.007
	FFN-ECFP	0.921 ± 0.005
	AA-MPNN-CL (without ECFP)	0.938 ± 0.008
	AA-MPNN-CL (with ECFP)	0.951 ± 0.006
Caco-2 permeability	RF-ECFP	0.869 ± 0.013
	SVM-ECFP	0.874 ± 0.011
	FFN-ECFP	0.885 ± 0.011
	AA-MPNN-CL (without ECFP)	0.904 ± 0.019
	AA-MPNN-CL (with ECFP)	0.919 ± 0.019

### Web service for property prediction powered by Enalos cloud platform

The predictive models developed in this study are readily accessible to the public for use and validation through the Enalos Cloud Platform. This web-based service hosts two key models: the Blood–Brain Barrier permeability model and the Caco-2 cell permeability model. These models are available at the following URLs:

- BBB permeability model: Enalos Cloud BBB Permeability
- Caco-2 cell line permeability: Enalos Cloud Caco-2 Permeability

The Enalos Cloud Platform is specifically designed to support researchers and professionals in the pharmaceutical and biochemical sectors by providing a robust tool for the computational prediction of molecular permeability. The platform enables the calculation of permeability for untested molecules, facilitating the process of drug discovery and development with high efficiency and accuracy. All models presented in this study were trained on the NVIDIA DGX Station, a high-performance AI workstation with four NVIDIA Tesla V100 GPUs. Moreover, the Enalos Cloud Platform itself is hosted and running on the NVIDIA DGX Station, leveraging its computational power to deliver real-time predictions.

**Fig. 7:**
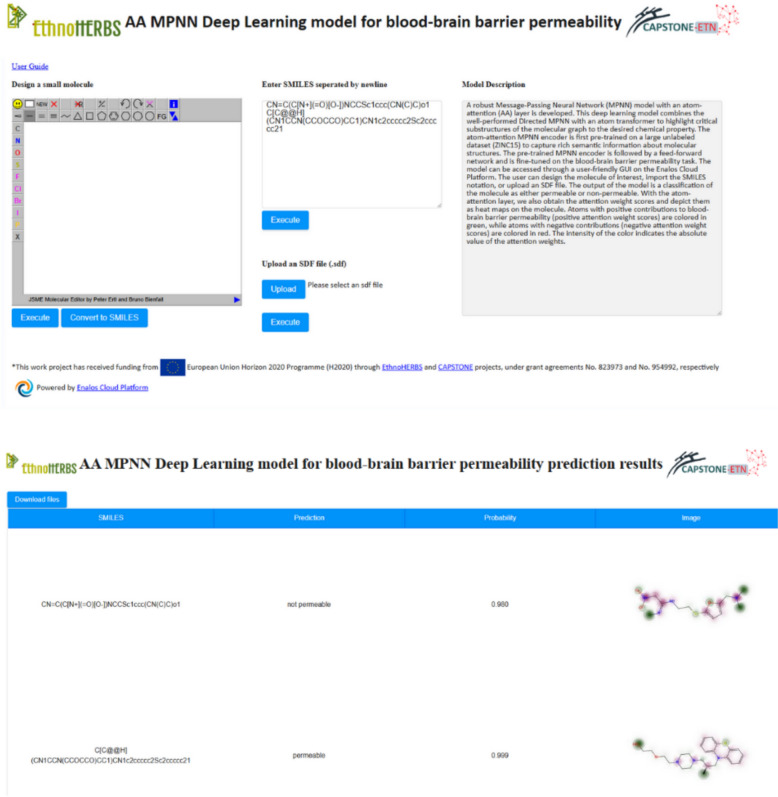
User-interface and results page for the Atom-Attention (AA) MPNN model for BBB permeability model

**Fig. 8:**
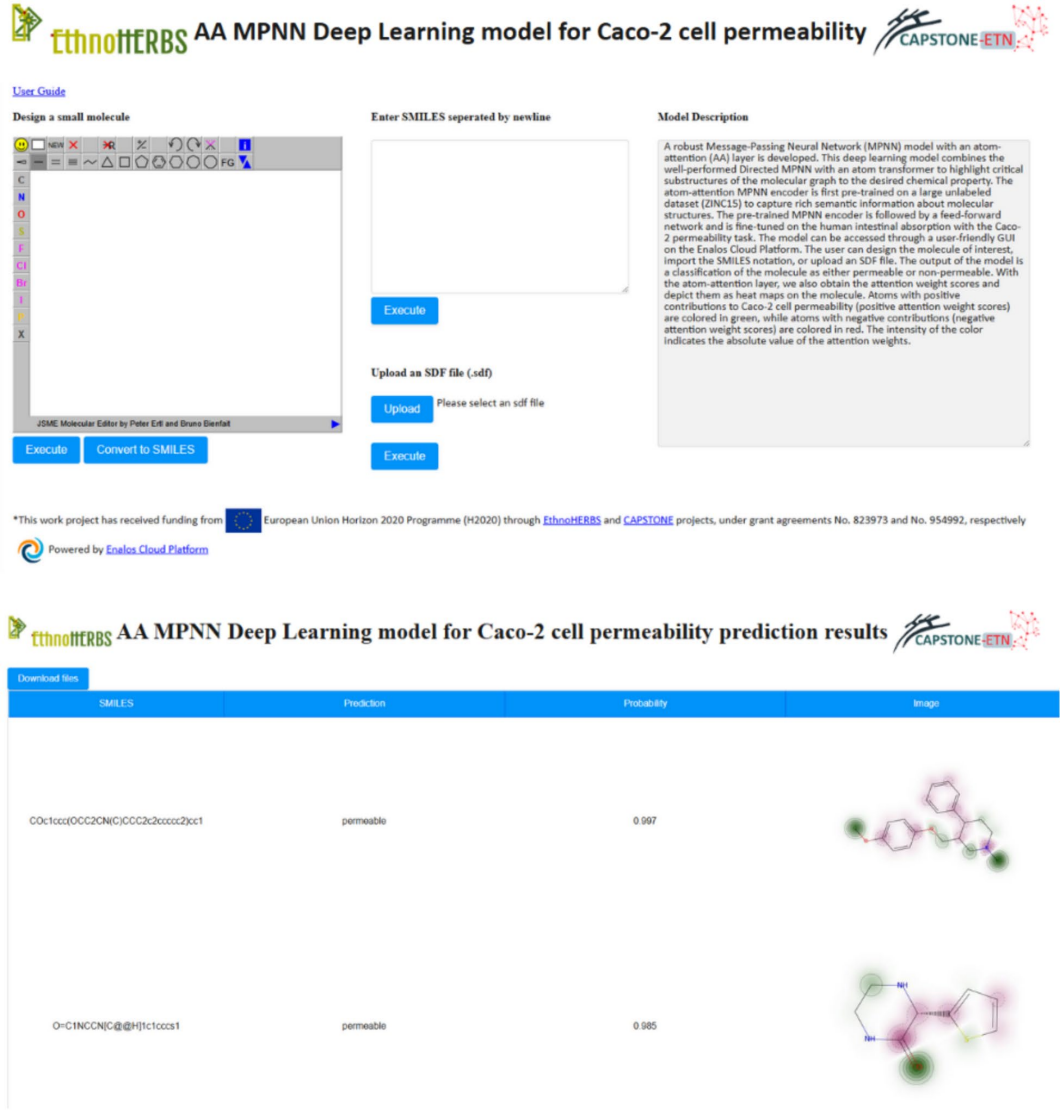
User-interface and results page for Αthe Atom-Attention (AA) MPNN model for Caco-2 cell permeability model

The user interface of the Enalos Cloud Platform is designed to ensure ease of use, making advanced computational tools accessible even to users without extensive technical or programming knowledge (Figs. 7,  8). Researchers can input molecular structures in several formats:

- By entering the SMILES notation directly into the platform.
- By drawing the chemical structure using an integrated molecular drawing tool.
- By uploading a structure data file (.SDF) containing the molecular information.

Upon submission of the molecular information, the platform processes the input data to predict whether a compound is likely to be permeable or non-permeable across the BBB or Caco-2 cell barrier. The results are provided in a matter of seconds, displaying not only the permeability status but also a visual representation of the molecule. This visualization includes a heatmap coloring to indicate the atom attention (AA) weights, offering insights into which parts of the molecule most significantly impact its predicted permeability. This feature, as outlined in the "Interpretability and Visualization" section below, is invaluable for researchers seeking to understand the molecular basis of the model's predictions, enhancing both the interpretability and applicability of the results. The Enalos Cloud Platform thus serves as a critical tool in streamlining the evaluation of molecular properties, significantly reducing the time and resources typically required for such activities. By integrating advanced predictive models with user-friendly interfaces, the platform democratizes access to cutting-edge computational predictions and supports the broader scientific community in advancing drug design and chemical research.

### Interpretability and visualization

The ability to interpret the results from complex DL models is crucial. Most DL models are considered "black boxes" because they provide limited insight into how they predict the properties of compounds, and which molecular substructures contribute significantly to the final predictions. This lack of transparency can hinder the broader acceptance of and trust in the results these models produce. In our AA-MPNN model, however, we enhance interpretability through the use of the atom-attention layer, which allows us to access attention weight scores. These scores highlight the specific interactions and importance of various molecular substructures in relation to the predicted outcomes, in this case barrier permeability. By investigating the latent linkages between these substructures and the predicted endpoint, insights into the molecular mechanics that drive the model’s decisions are obtained. Furthermore, employing color-coded heat maps for each molecule simplifies the visualization of these atomic attention weights. This visualization technique makes it straightforward to identify which parts of the molecule are crucial for determining drug permeability. For instance, areas highlighted with more intense colors in the heat map indicate regions of the molecule that have a stronger influence on the model's predictions. This not only aids in understanding the model’s function but also provides valuable insights into the pharmacokinetic properties of the compounds, such as their ability to penetrate biological barriers.

**Fig. 9:**
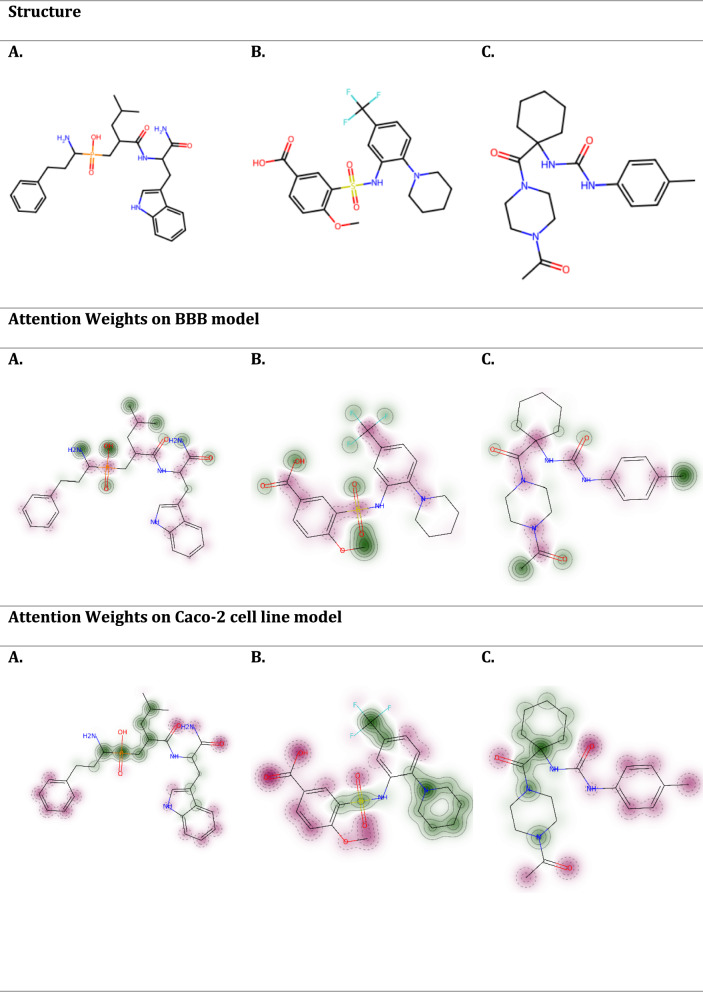
Visualization of the atom attention weights of three selective inhibitors of ERAP1. Atoms with positive contribution to permeability are colored in green, while atoms with negative contribution are colored in red. The intensity of the color indicates the absolute value of the attention weights. **A** DG013A, **B** 4-methoxy-3-(N-(2-(piperidin-1-yl)-5-(trifluoromethyl) phenyl)sulfamoyl)benzoic acid, **C** (1-(1-(4-acetylpiperazine-1-carbonyl)cyclohexyl)-3-(p-tolyl)urea

For our study, we selected a critical therapeutic target in order to evaluate the permeability of its inhibitors and to visualize the outcomes. The target in question is the Endoplasmic Reticulum Aminopeptidase 1 (ERAP1) protein, known for its aminopeptidase activity, which plays a pivotal role as a "molecular ruler" in shaping the major histocompatibility complex I (MHC I) immunopeptidome. ERAP1 is implicated in various autoimmune and autoinflammatory conditions, including Ankylosing Spondylitis, Inflammatory Bowel Disease, Psoriasis, and certain cancer types [52]. This association makes ERAP1 a significant point of interest in therapeutic research. To investigate the effectiveness of the model for predicting permeability, we used three selective inhibitors of ERAP1 namely DG013A which is a phosphinic acid tripeptide mimetic, 4-methoxy-3-(N-(2-(piperidin-1-yl)-5-(trifluoromethyl)phenyl) sulfamoyl) benzoic acid, and (1-(1-(4-acetylpiperazine-1-carbonyl)cyclohexyl)-3-(p-tolyl)urea [53, 54], as probes to study their permeability properties (Fig. 9). These inhibitors are crucial for understanding how impacting ERAP1 activity affects disease mechanisms and for evaluating the potential side effects and efficacy of ERAP1-targeted therapies in clinical settings. By analyzing the permeability of these inhibitors, we can gain insights into their ability to reach and inhibit the ERAP1 enzyme within the human body, which is essential for their effectiveness as therapeutic agents.

**Fig. 10:**
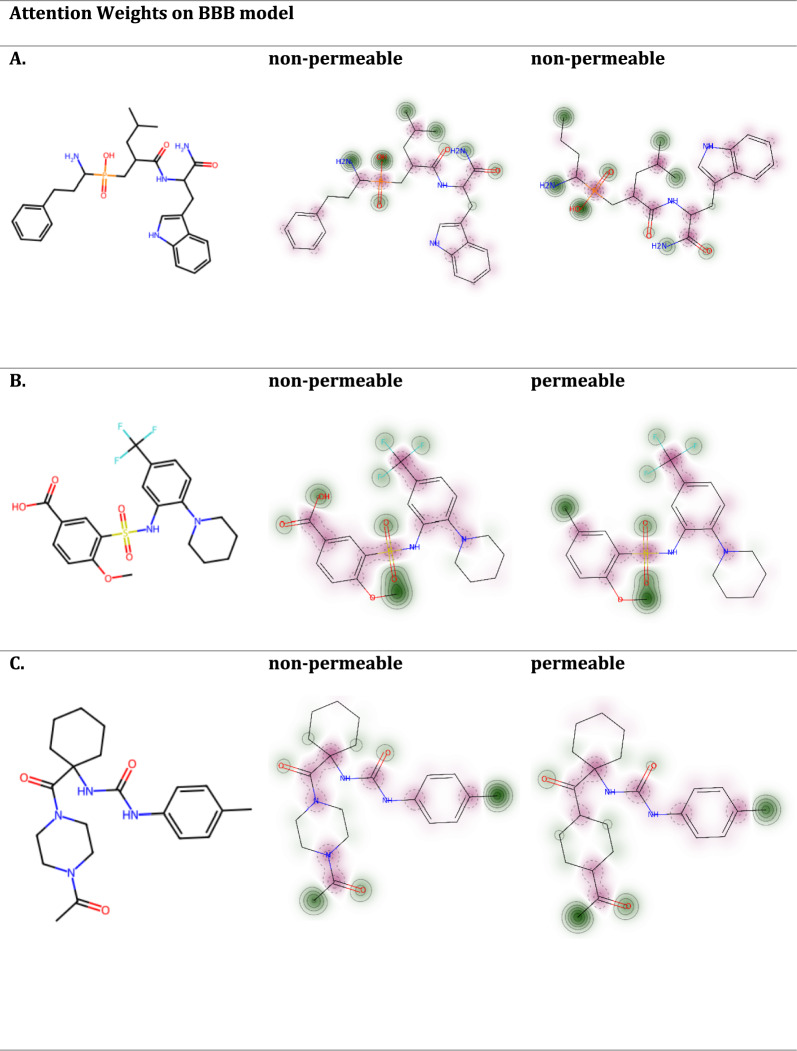
Modifications of initial molecular structures of the ERAP1 enzyme inhibitors to design permeable compounds

In this study, it was noted that the atom-attention layer of the model is discriminating in terms of its focus on various molecular substructures depending on the specific downstream molecular property prediction task. This adaptive focusing is particularly evident in how different functional groups are highlighted in the model predictions. For example, sulfonamide groups are consistently highlighted as having a negative impact on permeability in the BBB model. This observation is supported by literature [55], which notes that sulfonamide groups significantly reduce permeability due to their chemical properties. Benzene rings are generally associated with negative contributions to permeability predictions, while cyclohexane usually exhibits a positive influence, enhancing permeability across barriers in most scenarios. For the BBB permeability predictions, the AA MPNN model identified inhibitors A, B and C as low-permeable. To improve the permeability of the non-permeable compounds, we explored structural modifications, which are illustrated in Fig. 10. For inhibitor A, removing the benzene ring increases permeability. For inhibitor B, replacing the polar carboxyl group with a non-polar, hydrophobic methyl group altered its permeability profile, increasing its predicted permeability from non-permeable to more permeable. This change suggests that the carboxyl group's polarity might hinder its ability to cross the lipid-rich BBB membrane, while the methyl group enhances the compound's overall lipophilicity, facilitating its transit. In the case of inhibitor C, substituting pyrazine with cyclohexane resulted in a more lipophilic compound with a reduced molecular weight, which positively affected its permeability characteristics. This modification highlights how small changes in molecular structure can significantly impact a drug's ability to penetrate biological barriers.

### Validation of models on compounds from the literature

To further validate our models externally, we conducted a literature search to identify compounds with well-documented experimental permeability data that were not included in our training dataset. For example, varenicline is a partial agonist of the nicotinic acetylcholine receptor and its known to cross the BBB effectively [56], while nicotine rapidly crosses the BBB due to its small size and lipophilicity, leading to fast central nervous system effects [57]. Our model correctly classified both compounds as permeable. On the other hand, dopamine, which cannot efficiently cross the BBB in its native state due to its polarity, was correctly predicted as a low permeable compound [58]. In contrast, its prodrug, levodopa, which utilizes transporters to cross the BBB, was predicted as permeable [59]. Levodopa, modified by β-carboxylation to generate an amino acid backbone, crosses the BBB with this modification enhancing its permeability as shown in Supplementary Information Table S6.

For intestinal absorption prediction, our model correctly predicted antipyrine as permeable, which is a small lipophilic molecule with high permeability across intestinal barrier [60]. Similarly, caffeine, which is in class I of the Biopharmaceutics Classification System and has excelent intestinal absorption, was also predicted as permeable. Acyclovir, a dopamine antagonist, has low intestinal permeability and low oral bioavailability. Our prediction of low permeability comes in agreement with known data, as it exhibit poor passive absorption and aligns with the literature. These validation results (Supplementary Information Table S6 and S7) demonstrate that our models effectively distinguish between high- and low-permeability compounds, with predictions aligning well with established experimental data.

## Conclusions

In this study, we have developed a sophisticated message-passing framework that leverages CL to enhance the traditional molecular property prediction process. This framework incorporates both additive and scaled dot-product attention mechanisms at the atomic level, enabling our atom-attention MPNN model to focus more precisely on critical molecular features that drive the desired behaviour, in this case barrier crossing. Combined with CL, this approach has yielded significant improvements in predictive accuracy, particularly for BBB permeability and human intestinal permeability prediction, two key ADMET properties that influence drug absorption and CNS penetration.

By pretraining the atom-attention MPNN on a large, unlabeled dataset, the model has been able to learn robust and comprehensive molecular representations. This extensive pretraining allows the model to effectively generalize across the vast chemical space, a critical factor in its improved performance relative to the non-pretrained model. The use of self-attention mechanisms plays a pivotal role in this context, as it enhances the model's ability to extract and emphasize molecular representations that are most relevant to the properties being predicted. This targeted focus aids in achieving more accurate and reliable prediction results.

A key aspect of our study involved the use of three selective inhibitors of the ERAP1 protein to test the model's effectiveness. The results demonstrated how self-attention mechanisms can significantly enhance the model's interpretability by clearly highlighting the impact of specific molecular substructures on barrier permeability. Notably, our findings revealed that different molecular substructures influence the ability of compounds to cross specific barriers, as evidence by the identification of different “driving” features for BBB and Caco-2 permeability. Furthermore, we validated our models on compounds found in the literature with experimental permeability data. These findings not only validate the model's predictive capabilities but also shed light on the underlying atomic interactions and contributions to the observed permeability outcomes.

To make these advanced computational tools more accessible to the broader scientific community, the two models have been deployed as web applications through the Enalos Cloud Platform. This online platform allows users to easily input molecular structures and receive permeability predictions in real-time, alongside visualization of the areas in the molecular structure that most affect barrier crossing and as such could act as sites for structural modification of the molecule to increase permeability. The web applications provide a user-friendly interface that requires no prior programming knowledge, thereby democratizing access to state-of-the-art predictive technologies for drug discovery.

## Supplementary Information

Supplementary material 1.

## Data Availability

The datasets and the scripts for pretraining and fine-tuning are publicly available on our GitHub repository: https://github.com/NovaMechanicsOpenSource/Atom-Attention-MPNN.git.

## Abbreviations

ADMET: Absorption, distribution, metabolism, excretion, and toxicity
BBB: Blood–brain barrier
CADD: Computer-assisted drug discovery
CL: Contrastive learning
CV: Cross-validation
D-MPNN: Directed message-passing neural network
DL: Deep learning
ECFP: Extended-connectivity fingerprints
ERAP1: Endoplasmic reticulum aminopeptidase 1
FFN: Feed-forward network
GCL: Graph contrastive learning
GCNs: Graph convolutional networks
GNNs: Graph neural networks
MACCS: Molecular access system fingerprints
MCC: Matthew’s correlation coefficient
ML: Machine learning
MPNN: Message-passing neural network
QSAR: Quantitative structure–activity relationship
QSPR: Quantitative structure–property relationship
RF: Random forest
RMSE: Root mean squared error
ROC-AUC: Area under the receiver operating characteristic curve
SMILES: Simplified Molecular Input Line Entry System
SSL: Self-supervised learning
SVM: Support vector machines
t-SNE: T-distributed stochastic neighbor embedding
