# Supplementary material for: Prediction of blood–brain barrier and Caco-2 permeability through the Enalos Cloud Platform: combining contrastive learning and atom-attention message passing neural networks
Source: J Cheminform. 2025 May 5;17:68. doi: 10.1186/s13321-025-01007-2 (PMC12051285; doi:10.1186/s13321-025-01007-2)
Supplement: Supplementary file 1 — Supplementary material 1. [file 13321_2025_1007_MOESM1_ESM.docx]

# Supplementary Information

# Prediction of blood-brain barrier and Caco-2 permeability through the Enalos Cloud Platform: combining contrastive learning and atom-attention message passing neural networks

Nikoletta-Maria Koutroumpa^1,2,3^, Andreas Tsoumanis^1^, Haralambos Sarimveis^2^, Iseult Lynch^4^, Georgia Melagraki^5^, Antreas Afantitis^1,3,6,*^

^1^ NovaMechanics Ltd., Nicosia 1070, Cyprus

^2^ School of Chemical Engineering, National Technical University of Athens, 157 80 Athens, Greece

^3^ Entelos Institute, Larnaca 6059, Cyprus

^4^ School of Geography, Earth and Environmental Sciences, University of Birmingham, Birmingham, UK

^5^ Division of Physical Sciences & Applications, Hellenic Military Academy, 166 73 Vari, Greece

^6^ NovaMechanics MIKE., 185 45 Piraeus, Greece

* Correspondence: afantitis@novamechanics.com

## Details of Molecular Datasets

Table S1 summarizes all datasets used in our work. We used a large unlabeled dataset from ZINC15 for pretraining. Datasets BBB and Caco-2 cell line consist of compounds and a class indicating if the compound is permeable or not of blood-brain barrier and human intestine, respectively. These are used to train a molecular property prediction model.

Table S1: Summary of all datasets for pretraining and fine-tuning for downstream tasks used in this work.

| Dataset | # Molecules | # Tasks | Task type | Metric | Available |
| --- | --- | --- | --- | --- | --- |
| ZINC15 | 250,000 | 0 | Unlabeled | NT-Xent loss | [1] |
| BBB | 7,807 | 1 | Classification | ROC-AUC | [2] |
| Caco-2 cell line | 1,827 | 1 | Classification | ROC-AUC | [3] |

### Caco-2 cell line permeability

In addition to k-Means clustering, we also performed Gaussian Mixture Modeling (GMM) with four Gaussian components to categorize low permeability, low/moderate, moderate/high and high permeability. Interestingly, the threshold identified by k-Means (logPapp=-5.5) aligns well with this categorization and the available dataset, providing a reasonable approximation for distinguishing between low and high permeability. Given the fact that there are several definitions of Caco-2 permeability cutoff, our data-driven clustering approach provides an alternative, unbiased way to define permeability classes while maintaining consistency with literature-based cutoffs.


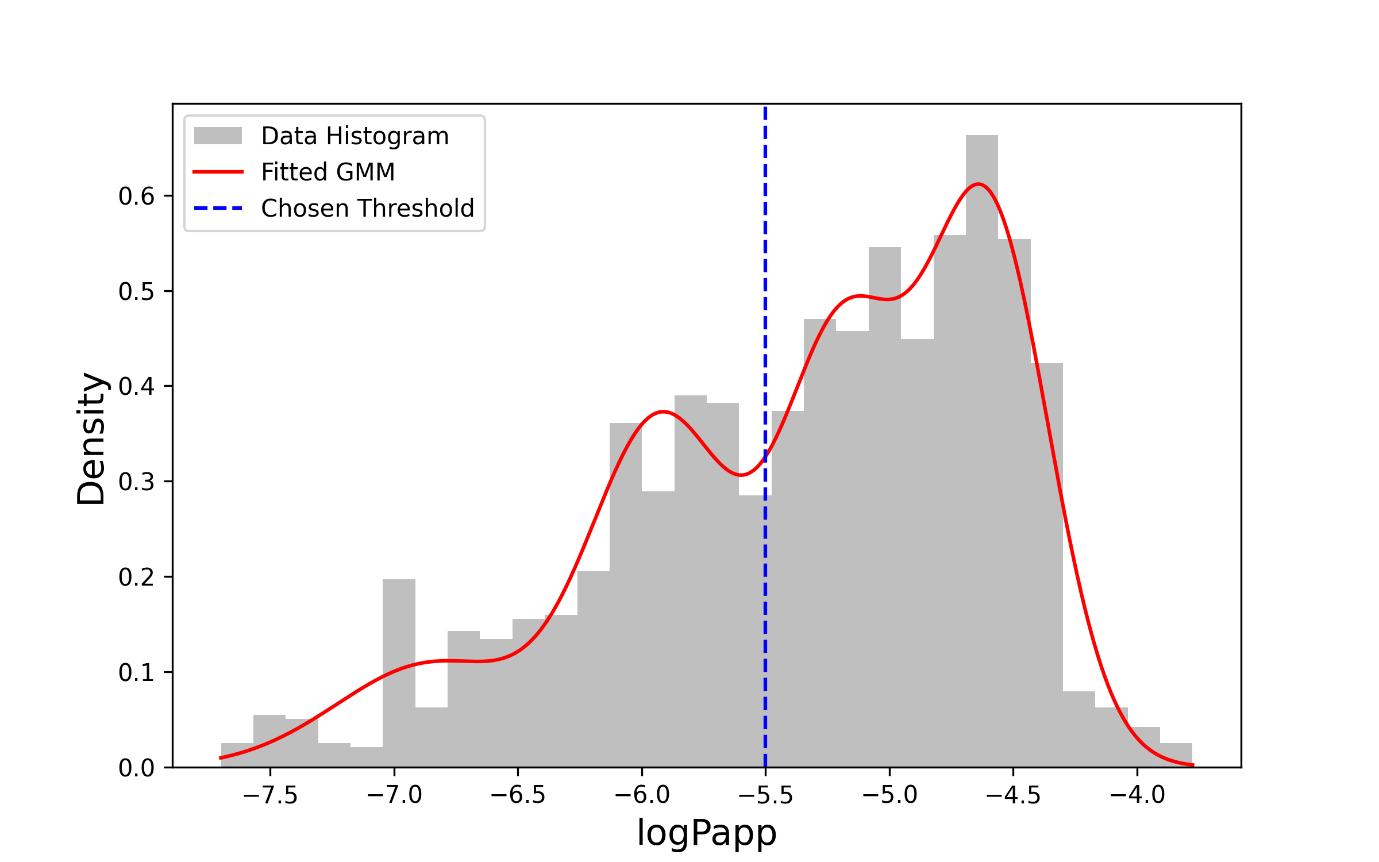


Figure S1: Distribution of logPapp values, Gaussian Mixture Modeling, and the threshold identified by k-Means clustering.

### ZINC15 pretraining dataset


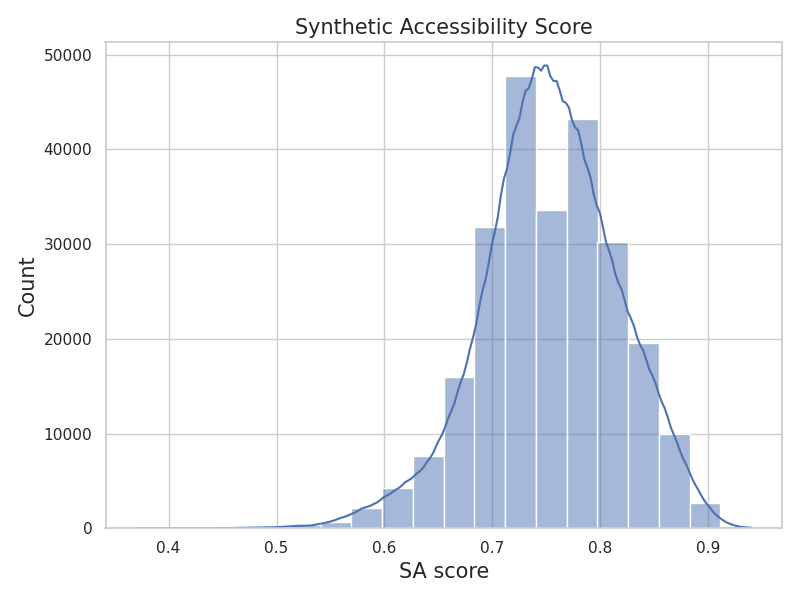


Figure S2: Synthetic accessibility score (SA score) of ZINC15 dataset. The SA score values are normalized in range [0,1]. A higher SA score indicates a more synthesizable molecule.


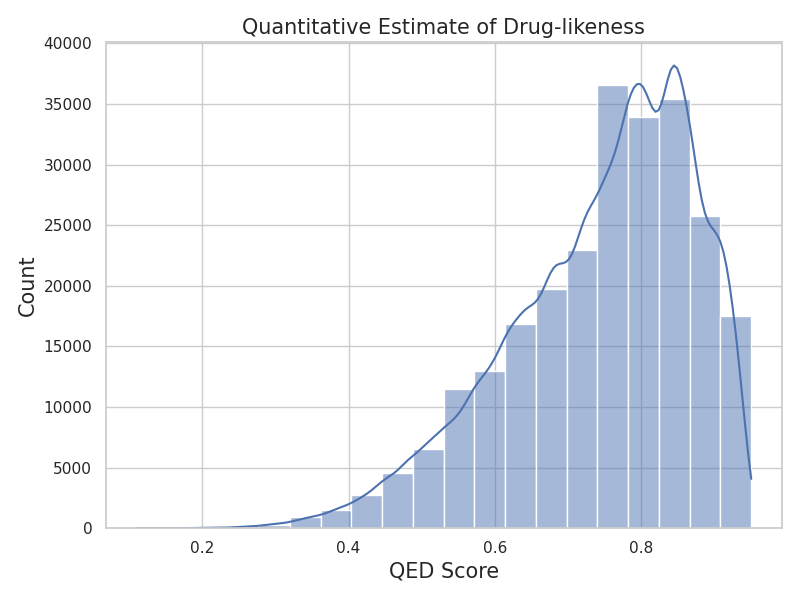


Figure S3: Quantitative Estimate of Drug-likeness (QED) score of ZINC15 dataset. A higher QED score indicates a more drug-like molecule.

Table S2: Details of ZINC15 dataset used for pretraining.

| Dataset | # Molecules | Mean SA Score | Mean QED Score | # Unique Scaffolds |
| --- | --- | --- | --- | --- |
| ZINC15 | 250,000 | 0.75 | 0.74 | 138,768 |


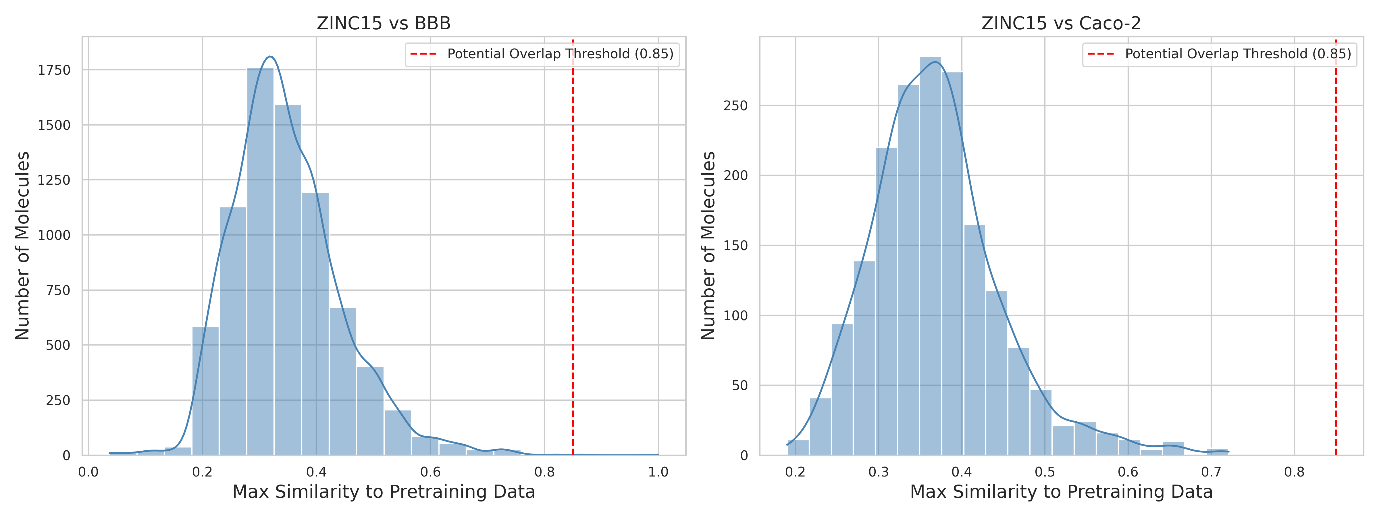


Figure S4: Maximum similarities between BBB dataset and ZINC15 and between Caco-2 cell line dataset and ZINC dataset. We set a similarity threshold of 0.85 to show there is no overlap between downstream dataset and pretraining dataset.

## Featurization

We followed Liu et al. [6] approach to build the set of node and bond features to embed the two-dimensional molecular graph. SMILES are converted to 2D graphs using RDKit. The details of node and bond features can be found in Table S3 and Table S4, respectively.

Table S3: Atom features included as nodes in the Knowledge Graph representation of a molecule for the message passing phase of the MPNN.

| Feature | Description | Size |
| --- | --- | --- |
| *Atom type* | Type of atom, by atomic number | 100 |
| *Degree* | Number of bonds the atom is involved in | 6 |
| *Formal charge* | Electronic charge assigned to atom | 5 |
| *Chirality* | R, S, unspecified, unrecognized chirality | 4 |
| *Number of bonded hydrogens* | Number of bonded hydrogen atoms | 5 |
| *Hybridization* | sp, sp^2^, sp^3^, sp^3^d, sp^3^d^2^ | 5 |

Table S4: Bond features included as edges in the Knowledge Graph representation of a molecule for the message passing phase of the MPNN.

| Feature | Description | Size |
| --- | --- | --- |
| *Bond type* | Single, double, triple, or aromatic | 4 |
| *Ring* | Whether the bond is in ring | 1 |
| *Conjugated* | Whether the bond is conjugated | 1 |
| *Stereochemistry* | Bond’s stereochemistry (none, any, Z, E, cis, or trans) | 6 |

## Evaluation metrics

Table S5: Accuracy, precision, sensitivity and specificity

| *Evaluation metrics* |
| --- |
| $\boldsymbol{Accuracy=}\frac{\boldsymbol{TP+TN}}{\boldsymbol{TP+TN+FP+FN}}$ |
| $\boldsymbol{Precision=}\frac{\boldsymbol{TP}}{\boldsymbol{TP+FP}}$ |
| $\boldsymbol{Sensitivity=TPR=}\frac{\boldsymbol{TP}}{\boldsymbol{TP+FN}}$ |
| $\boldsymbol{Specificity= TNR=}\frac{\boldsymbol{TN}}{\boldsymbol{TN+FP}}$ |

where:

- TP (True Positives) are correctly predicted positive values.
- TN (True Negatives) are correctly predicted negative values.
- FP (False Positives) are incorrectly predicted as positive.
- FN (False Negatives) are incorrectly predicted as negative.

## Detailed Results of cross-validation

**
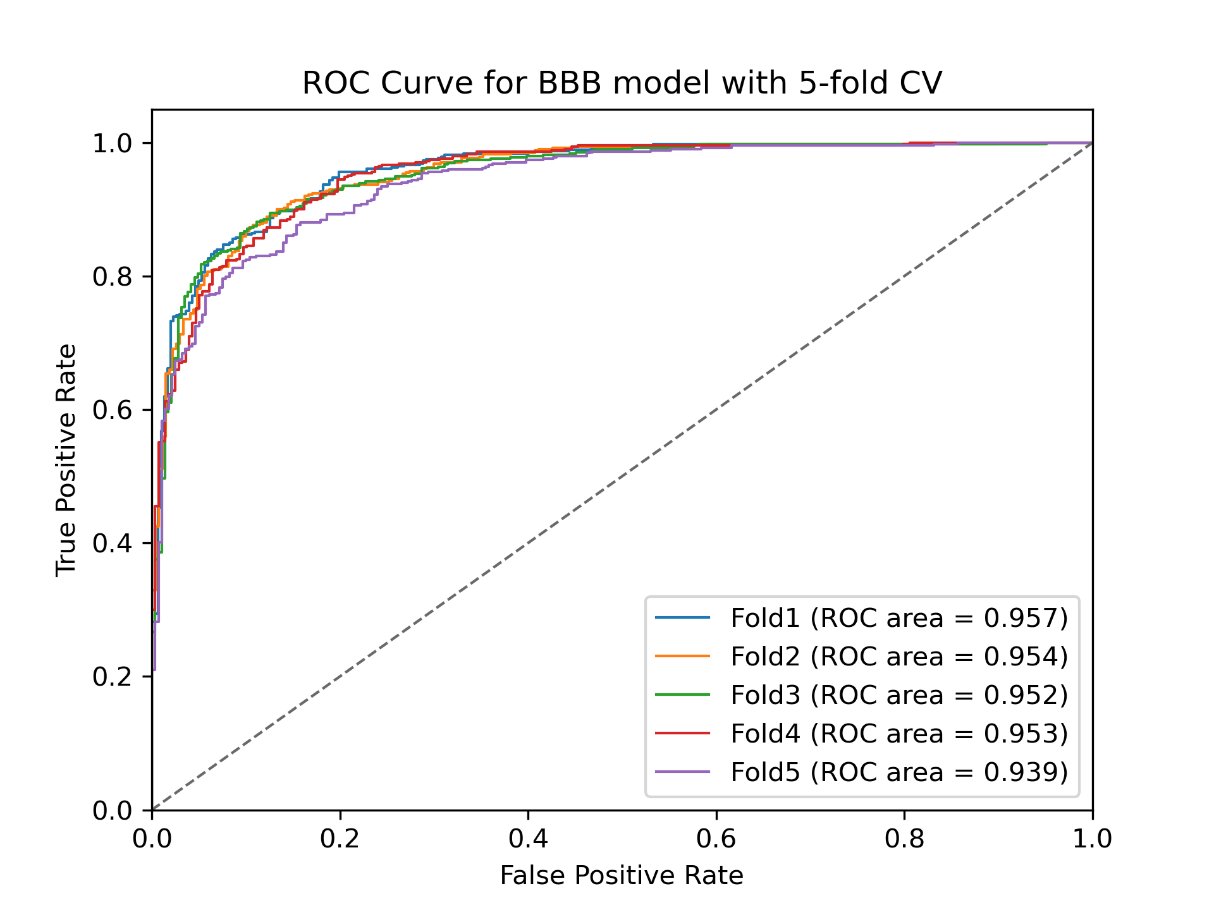
**

Figure S5: ROC curves of five folds in cross validation for BBB model. MPN is pre-trained on ZINC15 database.


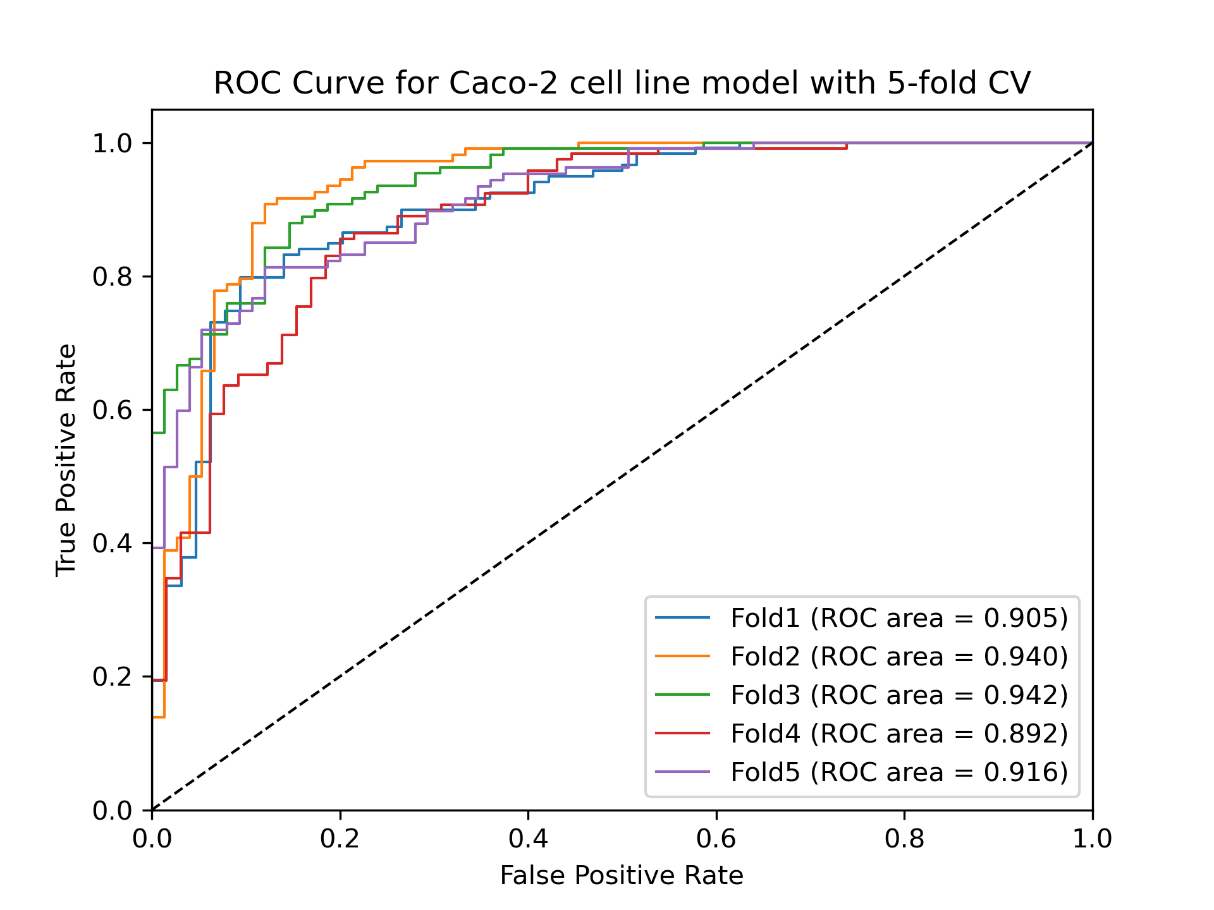


Figure S6: ROC curves of five folds in cross validation for Caco-2 cell line model. MPN is pre-trained on ZINC15 database.

## Validation of models on compounds from the literature

Table S6: Blood brain barrier permeability prediction of known compounds.

| Name | BBB Class | BBB prediction | |
| --- | --- | --- | --- |
|  |  | class | Attention Weights |
| Varenicline | High permeability | High permeability | 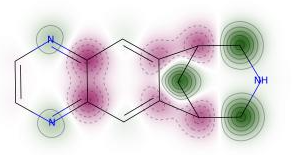 |
| Nicotine | High permeability | High permeability | 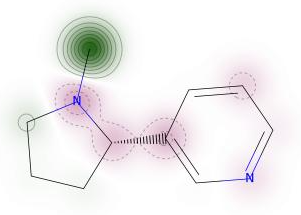 |
| Levodopa | High permeability | High permeability | 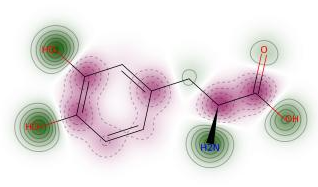 |
| Dopamine | Low permeability | Low permeability | 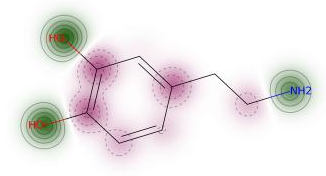 |

Table S7: Caco-2 permeability prediction of known compounds.

| Name | Caco-2 Class | Caco-2 prediction | |
| --- | --- | --- | --- |
|  |  | class | Attention Weights |
| Antipyrine | High permeability | High permeability | 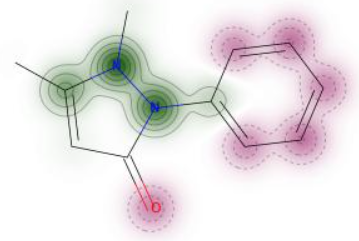 |
| Caffeine | High permeability | High permeability | 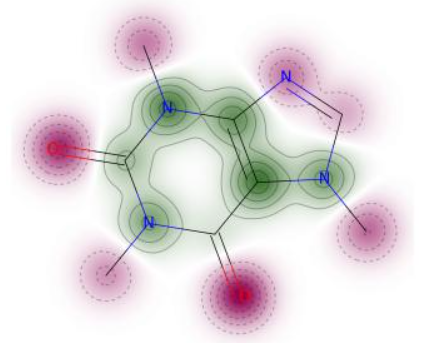 |
| Acyclovir | Low permeability | Low permeability | 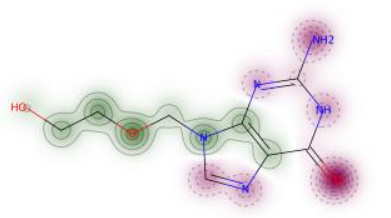 |

## References

[1] T. Sterling and J. J. Irwin, “ZINC 15 – Ligand Discovery for Everyone,” *J. Chem. Inf. Model.*, vol. 55, no. 11, pp. 2324–2337, Nov. 2015, doi: 10.1021/acs.jcim.5b00559.

[2] F. Meng, Y. Xi, J. Huang, and P. W. Ayers, “A curated diverse molecular database of blood-brain barrier permeability with chemical descriptors,” *Sci. Data*, vol. 8, no. 1, p. 289, Oct. 2021, doi: 10.1038/s41597-021-01069-5.

[3] Y. Wang and X. Chen, “QSPR model for Caco-2 cell permeability prediction using a combination of HQPSO and dual-RBF neural network,” *RSC Adv.*, vol. 10, no. 70, pp. 42938–42952, 2020, doi: 10.1039/D0RA08209K.

[4] H. Pham The *et al.*, “In Silico Prediction of Caco‐2 Cell Permeability by a Classification QSAR Approach,” *Mol. Inform.*, vol. 30, no. 4, pp. 376–385, Apr. 2011, doi: 10.1002/minf.201000118.

[5] G. Falcón-Cano, C. Molina, and M. Á. Cabrera-Pérez, “Reliable Prediction of Caco-2 Permeability by Supervised Recursive Machine Learning Approaches,” *Pharmaceutics*, vol. 14, no. 10, p. 1998, Sep. 2022, doi: 10.3390/pharmaceutics14101998.

[6] C. Liu, Y. Sun, R. Davis, S. T. Cardona, and P. Hu, “ABT-MPNN: an atom-bond transformer-based message-passing neural network for molecular property prediction,” *J. Cheminformatics*, vol. 15, no. 1, p. 29, Feb. 2023, doi: 10.1186/s13321-023-00698-9.
